# Multi-Fault Diagnosis of Gearbox Based on Improved Multipoint Optimal Minimum Entropy Deconvolution

**DOI:** 10.3390/e20080611

**Published:** 2018-08-17

**Authors:** Fuhe Yang, Xingquan Shen, Zhijian Wang

**Affiliations:** School of Mechanical Engineering, The North University of China, Taiyuan 030051, China

**Keywords:** improved multipoint optimal minimum entropy deconvolution, multi-fault, fault diagnosis

## Abstract

Under complicated conditions, the extraction of a multi-fault in gearboxes is difficult to achieve. Due to improper selection of methods, leakage diagnosis or misdiagnosis will usually occur. Ensemble Empirical Mode Decomposition (EEMD) often causes energy leakage due to improper selection of white noise during signal decomposition. Considering that only a single fault cycle can be extracted when MOMED (Multipoint Optimal Minimum Entropy Deconvolution) is used, it is necessary to perform the sub-band processing of the compound fault signal. This paper presents an adaptive gearbox multi-fault-feature extraction method based on Improved MOMED (IMOMED). Firstly, EEMD decomposes the signal adaptively and selects the intrinsic mode functions with strong correlation with the original signal to perform FFT (Fast Fourier transform); considering the mode-mixing phenomenon of EEMD, reconstruct the intrinsic mode functions with the same timescale, and obtain several intrinsic mode functions of the same scale to improve the entropy of fault features. There is a lot of white noise in the original signal, and EEMD can improve the signal-to-noise ratio of the original signal. Finally, through the setting of different noise-reduction intervals to extract fault features through MOMED. The proposed method is compared with EEMD and VMD (Variational Mode Decomposition) to verify its feasibility.

## 1. Introduction

The gearbox in mechanical equipment is one of the most important power transmission components; its health directly affects whether the mechanical equipment can work normally. If we can accurately predict the faults’ location, the huge human and financial losses that are caused by faults can be effectively avoided, so research of new composite fault-diagnosis methods play a decisive role in the normal operation of the gearbox. When the inner and outer ring or the rolling body of the gear and bearings fails, the failures are coupled to each other, which makes faults often be represented in the form of compound faults, and periodic pulses will appear in vibration signals [1,2,3]. The compound fault feature extraction of rotating machinery is still a big challenge [4,5], especially in strong-noise environments. So we are still in need of a lot of research to solve how to extract compound fault features in strong-noise environments. In the aspect of compound fault diagnosis, Ensemble Empirical Mode Decomposition (EEMD) can self-adaptively resolve different feature components into different modal functions [6]. But, there is often modal aliasing in EEMD; so-called modal aliasing refers to the same intrinsic mode function (IMF) containing different feature components, or the same timescale is broken down in different IMFs, which further leads to entropy loss. Although EEMD improves the precision of the decomposition by adding different white noises to the original signal and repeatedly asking the mean of IMFs, the literature [7,8,9] confirmed EEMD is more effective and accurate to the fault feature extraction of rotating machinery; it can self-adaptively resolve a complex signal into several IMFs. If the signal frequency band is too wide or the signal–noise ratio (SNR) too low, it will affect the decomposition efficiency of the EEMD [10]. The reason is that decomposition accuracy is greatly affected by the level of added white noise. If the level of white noise selected is too large, it will lead to overdecomposition. Mode aliasing still exists while it is too small, but it will not be enough to change the distribution of extreme value points [11]. The literature [12] indicates that how to self-adaptively select the level of white noise has still not been resolved. Wang [13] optimize the choice of white noise in EEMD by the combined modal-function method and, at the same time, improve the efficiency of its decomposition, but through the analysis of the simulation signal and the measured signal, modal aliasing still can’t be avoided completely. To sum up the above analysis, EEMD has been successfully applied in fault diagnosis, but due to the inappropriate selection for white-noise levels, there is still modal aliasing, which leads to entropy losses; therefore, we put forward combined IMFs (CIMFs) so that the original signal can be self-adaptively resolved into a different frequency band when a white-noise level is given, by combining several adjacent IMFs with the same frequency band after being resolved by the EEMD.

In 2016, McDonald [14] proposed a Multipoint Optimal Minimum Entropy Deconvolution (MOMED) method for rotating machine fault extraction. It is an improvement of minimum entropy deconvolution, which is to solve the optimal filter with the kurtosis maximum as the objective function, but it can only highlight a few pulses, whereas MOMED can highlight more pulses. MOMED uses a target to define the location and weightings of the impulse train obtained by deconvolution. These target goals are very suitable for feature extraction of a vibration source of a rotating machine that generates a pulse per rotation. However, when multiple faults coexist or under strong background noise, it is difficult to accurately extract the fault period components due to the influence of more than one fault period or background noise. Therefore, it is necessary to pretreat the vibration signal. When multiple faults coexist, coupled with white-noise pollution, the frequency band of complex vibration signals is relatively wide, so it is difficult for traditional FFT to identify each fault feature, and it is likely to cause incorrect diagnosis. The EEMD can adaptively modulate the original signal. In addition, EEMD can separate signals in order of high and low frequencies. In particular, when multiple faults coexist, different timescales are decomposed into different intrinsic functions, and the failure frequency is determined by solving the IMF of each layer. However, a large number of experiments have shown that EEMD cannot completely separate different timescales, and there are serious modal aliasing, which results in entropy leakage. We can determine the IMF with the same fault characteristics in advance by using FFT in each layer of the IMF; we can separate the different frequency bands in original signals by CIMF, and these operations can not only decompose the original signal into CIMF with different scales, but also make the optimization of the parameters for MOMED noise reduction. The CIMF can adaptively divide the original signal into different frequency bands and improve the entropy of the IMF. The period of the fault pulse in each CIMF can be determined by FFT. Finally, the appropriate period interval is input and the fault feature is extracted by MOMED. This paper explores a new method of fault feature extraction based on IMOMED, which can accurately identify the fault characteristics of the gearbox, and provides a new idea for fault feature extraction of rotating machinery.

## 2. Background and New Method

### 2.1. Introduction of EEMD

To solve the problem of mode mixing, EEMD is introduced based on the statistical properties of white noise. The EEMD algorithm can be given as follows.

(1) Given x(t) is an original signal, add a random white noise signal nj(t) to x(t)

(1) xj(t) = x(t) + nj(t) 

where xj(t) is the noise-added signal, j = 1,2,3,…,M and M is the number of trial.

(2) Decompose $x_{j}\left(t\right)$ into *I* $\left(IMFs\right)C_{i,j}$(i = 1,2,3,…,I) using EMD method. Where $C_{i,j}$ denotes the ith IMF of the *j*th trial, and *I* is the number of IMFs.

(3) If j < M, then go to step (1) with j = j + 1. Repeat steps (1) and (2) repeatedly, but with different white-noise series each time.

(4) Calculate the ensemble means of corresponding IMFs of the decompositions as the final result:

(2) $$C_{i}=\left(\sum_{j=1}^{m}C_{i,j}\right)/M\;$$

where i = 1,2,3…,I.

(5) $C_{i}\left(i\;=\;1,2,3,\ldots ,I\right)$ is the ensemble mean of corresponding IMF of EEMD. 

### 2.2. Introduction of Multipoint Optimal Minimum Entropy Deconvolution

In 1984, Cabrelli [15] proposed a new norm for impulse deconvolution, called the norm, and proved the deconvolution problem geometrically. The D-norm deconvolution problem can solve filter coefficients by an exact noniterative process. In 2016, McDonald [14] proposed a deconvolution target of multiple impulses at known locations based on Multi D-Norm, allowing for periodic impulse train deconvolution target goals, and introduced this maximization problem as MOMED. 

Assuming that the collected response signal is as shown in Equation (3),

(3) y(n) = h(n)x(n) + e(n) 

where *e*(*n*) is the white noise, *x*(*n*) is the impulse train, *h*(*n*) is the transfer function, and *y*(*n*) is the collected vibration signal. The essence of the MOMED algorithm is to find a FIR filter that resets the input signal *y*(*n*) through the output signal *x*(*n*).

(4) $$\mathrm{Multi}\;D-\mathrm{Norm}\;=\;\mathrm{MDN}\;(\vec{y},\vec{t})\;=\;\frac{1}{\left\|\vec{t}\right\|}\frac{{\vec{t}}^{T}\vec{y}}{\left\|\vec{y}\right\|}\;$$

MOMED:

(5) $$\max\;\mathrm{MDN}\;(\vec{y},\vec{t})\;=\;\underset{\vec{f}}{\max}\frac{{\vec{t}}^{T}\vec{y}}{\left\|\vec{y}\right\|}\;$$

The expected output of impulse deconvolution is a continuous spike. Where the target vector, $\vec{t}$, is a constant vector that defines the location and weightings of the goal impulses to be deconvolved, for example, $\vec{t}\;=\;{\left[\begin{array}{cccccccccc} 0 & 0 & 0 & 1 & 0 & 0 & 0 & 1 & 0 & 0 \end{array}\right]}^{T}$. The target vector $\vec{t}$ will aim to deconvolve two impulses in the output signal: one impulse at *n* = 4 and the other at *n* = 8, which represent the maximum of entropy. This Multi D-Norm is normalized to between 0 and 1, where a value of 1 indicates that the optimal target solution was reached. With the optimal target solution, the fault period with different sampling rates can be extracted, and the period of different fault characteristics at the same sampling frequency can be identified. Therefore, the target vector $\vec{t}$ can be used to determine the separation and position of the impulse signal. We solve the extrema of Equation (5) by taking the derivative with respect the filter coefficients ($\vec{f}=f_{1},f_{2},\ldots ,f_{L}$):

(6) $$\frac{d}{d\vec{f}}\left(\frac{{\vec{t}}^{T}\vec{y}}{\left\|\vec{y}\right\|}\right)\;=\;\frac{d}{d\vec{f}}\frac{t_{1}y_{1}}{\left\|\vec{y}\right\|}\;+\;\frac{d}{d\vec{f}}\frac{t_{2}y_{2}}{\left\|\vec{y}\right\|}\;+\;,\ldots ,\;+\;\frac{d}{d\vec{f}}\frac{t_{N-L}y_{N-L}}{\left\|\vec{y}\right\|}\;$$

N represents the number of sampling points, and L represents the size of the filter.

As we know:

$$\frac{d}{d\vec{f}}\frac{t_{k}y_{k}}{\left\|\vec{y}\right\|}\;=\;{\left\|\vec{y}\right\|}^{-1}t_{k}{\vec{M}}_{k}\;-\;{\left\|\vec{y}\right\|}^{-3}t_{k}y_{k}X_{0}\vec{y}\;$$

$${\vec{M}}_{k}\;=\;\left[\begin{array}{c} x_{k+L-1}\\ x_{k+L-2}\\ \vdots \\ x_{k} \end{array}\right]\;$$

Therefore Equation (6) can be written:

(7) $$\frac{d}{d\vec{f}}\left(\frac{{\vec{t}}^{T}\vec{y}}{\left\|\vec{y}\right\|}\right)\;=\;{\left\|\vec{y}\right\|}^{-1}\left(t_{1}{\vec{M}}_{1}\;+\;t_{2}{\vec{M}}_{2}\;+\;,\ldots ,\;+\;t_{N-L}{\vec{M}}_{N-L}\right)\;-\;{\left\|\vec{y}\right\|}^{-3}{\vec{t}}^{T}\vec{y}X_{0}\vec{y}\;$$

With the simplification:

$$t_{1}{\vec{M}}_{1}\;+\;t_{2}{\vec{M}}_{2}\;+\;,\ldots ,\;+\;t_{N-L}{\vec{M}}_{N-L}\;=\;X_{0}\vec{t}\;$$

and solving for extrema by equating to $\vec{0}$, Equation (7) becomes:

$${\left\|\vec{y}\right\|}^{-1}X_{0}\vec{t}\;-\;{\left\|\vec{y}\right\|}^{-3}{\vec{t}}^{T}\vec{y}X_{0}\vec{y}\;=\;\vec{0}\;$$

that is:

$$\frac{{\vec{t}}^{T}\vec{y}}{{\left\|\vec{y}\right\|}^{2}}X_{0}\vec{y}\;=\;X_{0}\vec{t}\;$$

Since $\vec{y}\;=\;X_{0}^{T}\vec{f}$ and assuming ${\left(X_{0}X_{0}^{T}\right)}^{-1}$ exists:

(8) $$\frac{{\vec{t}}^{T}\vec{y}}{{\left\|\vec{y}\right\|}^{2}}\vec{f}\;=\;{\left(X_{0}X_{0}^{T}\right)}^{-1}X_{0}\vec{t}\;$$

Since multiples of $\vec{f}$ are also solutions to Equation (8), multiples of $\vec{f}\;=\;{\left(X_{0}X_{0}^{T}\right)}^{-1}X_{0}\vec{t}$ are solutions to the MOMEDA problem. This method can completely avoid the iterative operation, regardless of whether the cycle is the integer and the length of the filter on the impact of noise reduction. The multistage transmission gearbox has wide frequency distribution and a large number of fault cycles. The noise reduction effect of MOMEDA is affected by the noise-reduction interval. When multiple faults coexist, only one periodic shock can be extracted for each interval of noise reduction. The smaller the interval, the more accurate the search, so you need to set the noise-reduction interval in advance.

## 3. Multi-Fault Feature Recognition under Strong Noise

EEMD is an adaptive noise-reduction method, which can improve the SNR [3,4,5,6,7,8]. However, when the white-noise amplitude is chosen improperly, the same fault feature is decomposed into different intrinsic modal functions, resulting in the weakening of the fault characteristic entropy, especially since the weak component of the composite fault is more likely to disappear. In order to overcome mode aliasing and improve the entropy of the same modal function, the CIMF method was chosen to improve frequencies of the original signal [13]. The specific method is as follows:

1. Setting the two parameters of EEMD. If the added white-noise amplitude is small, the distribution of the extreme points cannot be changed, and the added noise is meaningless. The larger amplitude will have excessive influence on the original signal, and the correct decomposition result cannot be obtained. In this paper, white-noise amplitude of the EEMD was determined by comparing the SNR after reconstructing the normal components. The method was as follows:

Noise amplitude gradually increased from zero, and the step size is 0.01.

(1) Adding 100 times white noise with amplitudes to the simulated signal x;

(2) Decomposing the signal after adding noise with EEMD;

(3) Calculating the SNR after reconstructing the normal components;

(4) When the SNR reached the maximum, the corresponding noise amplitude was the best amplitude.

The number of integrations had little effect on the decomposition efficiency of EEMD. In this paper, it was set to 100.

2. Resolve the signals by EEMD, and the IMFs that have correlation with the original signals are determined by correlation coefficient; the correlation coefficient is defined as shown in Equation (9):

(9) $$\rho_{xy}\;=\;\frac{E\left[(x\;-\;\mu_{x})(y\;-\;\mu_{y}\right]}{\sigma_{x}\sigma_{y}}\;$$

The relevance threshold in the article is 0.3, which is greater than 0.3 for correlation, otherwise it is irrelevant.

3. Solve the modal function FFT of each layer, determine their central frequency, and recombine the frequency band of the same frequency or integer times. By combining the neighboring IMFs that contain the same frequency, we obtain the CMF as follows [13,16]: IMF1, IMF2 and IMF3 have the same fault feature, IMF4, and IMF5 have the same fault feature, IMF6 contains the same frequency.

(10) CIMF1 = IMF1 + IMF2 + IMF3 

(11) CIMF2 = IMF4 + IMF5 

(12) CIMF3 = IMF6 

4. Determine the frequency of each CIMF above and calculate the corresponding period. Now that you’ve determined the component of frequency, why do you have to calculate the period? First, the result of EEMD decomposition is self-adaptive, but after EEMD decomposition, there exist noises in each layer of IMFs, and the noises consist of two parts: one is contained in the original signal, and the other is the adding noise of EEMD algorithm. These have not been completely neutralized, so the feature extraction of CIMF in entropy concentration is required. After the signal is decomposed by EEMD, the signal will be decomposed into high-order modal functions from high frequency to low frequency. Each layer has a fixed center frequency. The center-frequency reciprocal is the time period. Multiplying the period by the sampling frequency is the fault period (sampling point). Different frequencies represent different periods. 

5. Set three different search intervals and use MOMED to extract the features of different fault cycles.

**Figure 1:**
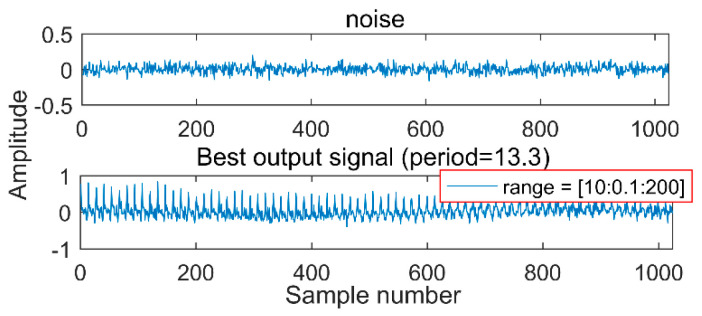
Noise and Multipoint Optimal Minimum Entropy Deconvolution (MOMED) noise-reduction results.

It should be noted that MOMED can continuously extract a series of periodic shocks, but it has the following characteristics:

(1) Given a random white-noise signal, a weak periodic pulse can be extracted by MOMED within a fixed noise-reduction interval, because the goal of MOMED is to solve the multipoint kurtosis in a series of signals and maximize them, then search for periodic pulses; there must be a result after each solution, and then it can extract the corresponding periodic pulses. As is shown in Figure 1, the original signal is a white noise.

After the noise reduction by MOMED, a 13.3 periodical pulse is extracted. Surprisingly, after running the program again, we would get another different periodic impact. In engineering applications, such results would lead to misdiagnosis. Therefore, a reasonable noise-reduction interval needs to be determined to ensure that every noise-reduction interval has an impact component. Regarding the selection method of the period interval, the article gives the simulation signal and explains accordingly. First, MOMED can only extract the continuous pulse of a single cycle, so it is necessary to decompose the signal and decompose different fault features into different IMFs. The selection of the cycle takes into account that, if the selected range is too large, a shock pulse that searches for half, 0.5, or 0.75 times of the cycle will occur, resulting in misdiagnosis. Therefore, the article selects a small interval as much as possible, but the lower limit of the interval is greater than 0.75 times. The upper limit of the interval is greater than one cycle, but if the overtaking is over, the amount of calculation increases.

**Figure 2:**
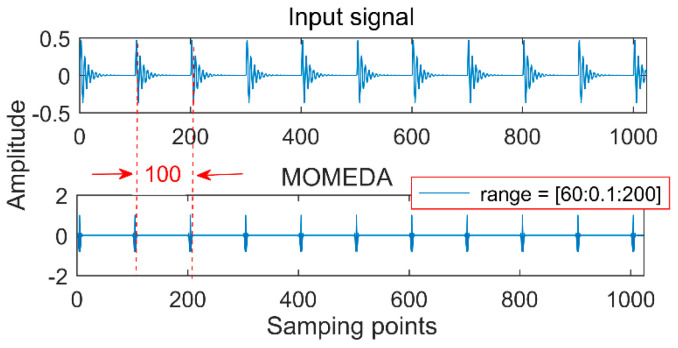
Periodic impact and MOMED noise reduction results.

**Figure 3:**
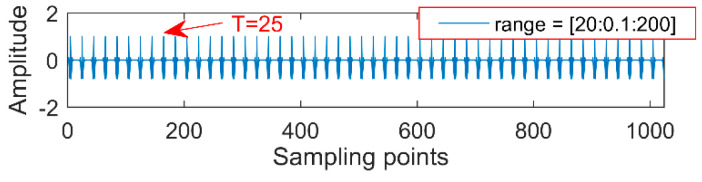
Different range of another impact noise component.

(2) An appropriate noise-reduction interval needs to be set each time. For example, a period of impact oscillation is 100 (the number of sampling points); if the noise-reduction interval is set in (60, 100) or (20, 200), extracting the cycle of 100 and 25 by MOMED is shown in Figure 2 and Figure 3, and there are multiples and factors of 100 in the large noise-reduction interval, so the components with a period of 25 are first extracted by MOMEDA. This result still leads to misdiagnosis, since the period becomes a quarter of the original signal and the corresponding frequency becomes four times, so it is necessary to reduce the period interval as much as possible to improve the noise reduction accuracy.

(3) For the complex vibration signal with multiple periodic pulses, such as the signal containing three cycles which are 50, 80, and 120, respectively, when it is given a noise interval (20, 150), perform the MOMED—perhaps we can only extract the impact signal whose cycle is 80. The reason is that the impact energy of 80 is strong and, at this time, MOMED defaults the periodic impact of 50 and 120 as noise. We will learn the specific instance analysis in the simulation signal for the next section.

(4) Based on the above analysis, when characteristic extraction of a complex multi-fault signal is carried out, the first step is to reduce its noise adaptively by EEMD. Secondly, it is necessary to determine that there is a fault period in each layer of the intrinsic function. Third, recombine the IMF of the same IMF; determine the impact period; and, finally, set different period ranges and MOMED noise reduction to further extract fault information. Considering that noise cannot be completely eliminated, it is necessary to reduce noise-reduction intervals as much as possible.

In order to improve the SNR, CIMF is used as the prefilter to reduce the noise, which not only reduces the interference of the background noise, but also increases the energy of the same frequency components. Then, the fault periods can be determined one by one through MOMED.

**Figure 4:**
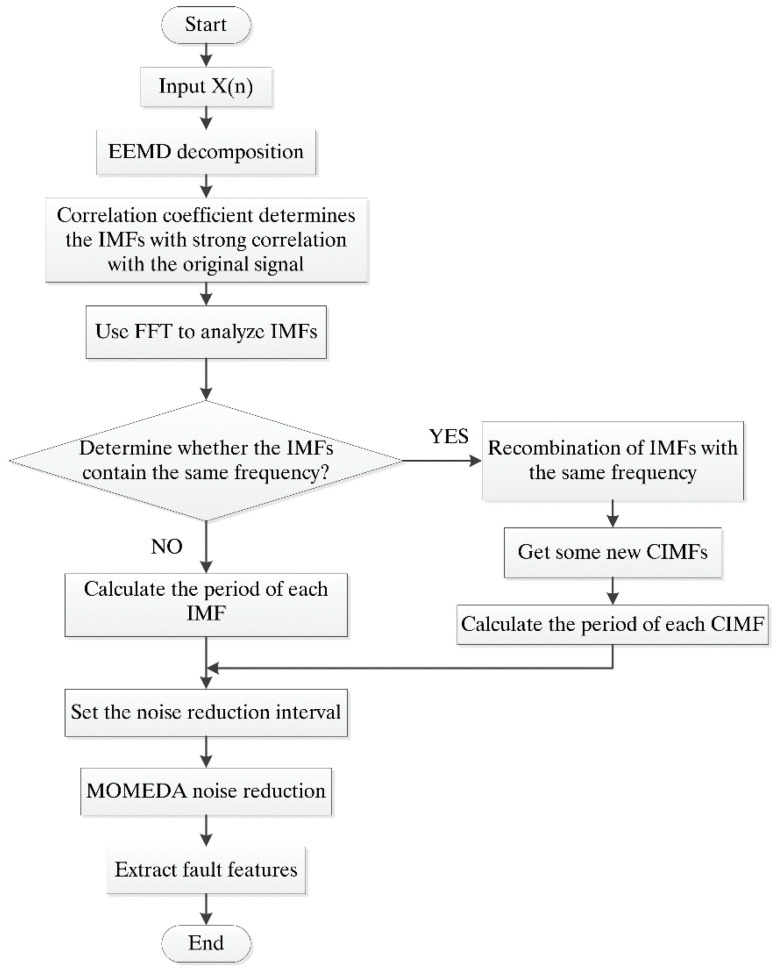
The flow chart of the proposed fault feature extraction method based on Improved Multipoint Optimal Minimum Entropy Deconvolution (IMOMED).

The flowchart of the proposed fault feature extraction method based on IMOMED is shown in Figure 4.

## 4. Performance Evaluation by Simulated Signals

**Figure 5:**
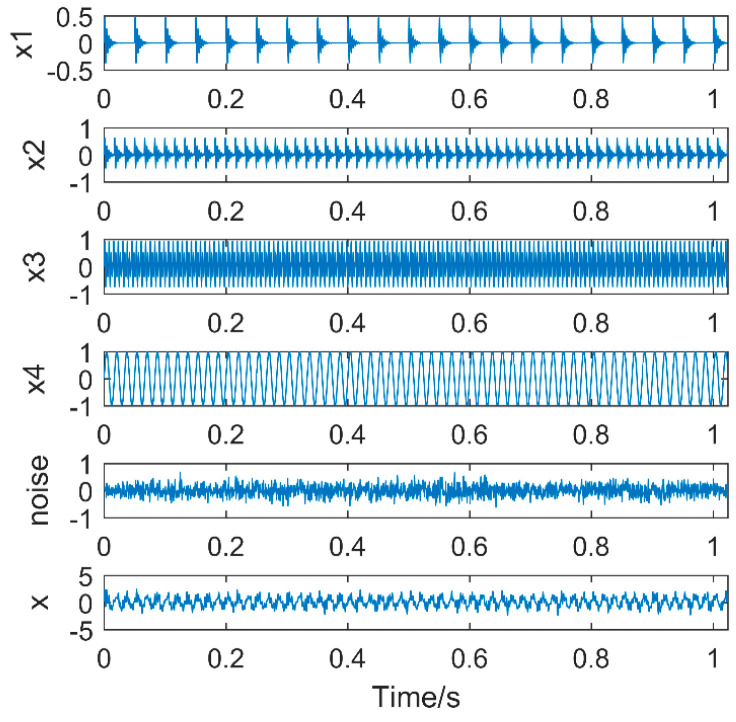
Simulation signal.

To evaluate the effectiveness of the proposed method to extract multiple faults, a typical multiple impact signal is simulated, which is shown in Figure 5. 

**Figure 6:**
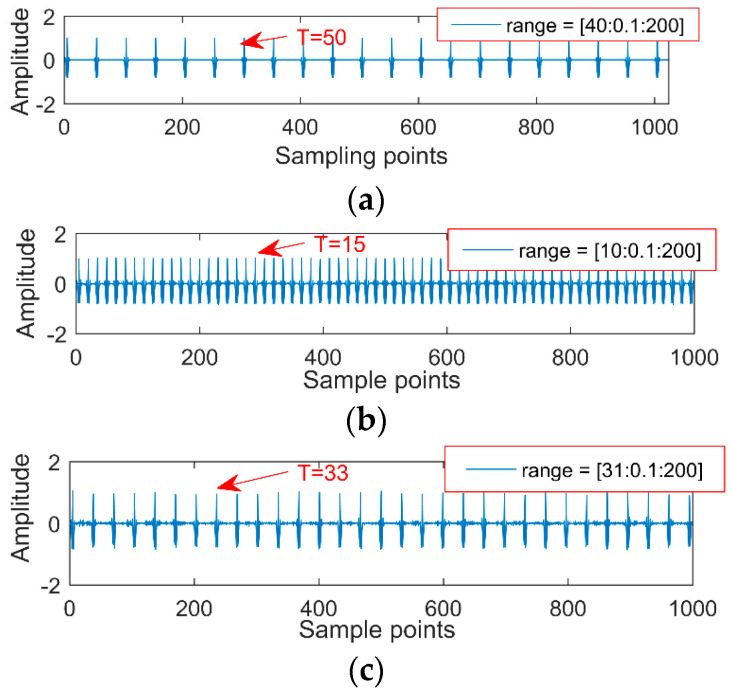
Different ranges after noise reduction by MOMED. (**a**) range = (40, 120), (**b**) range = (10, 200), (**c**) range = (31, 200).

Sampling points are 2048 and the sampling frequency is 2000 Hz, the simulation signal contains the noise signal (amplitude is 0.5), the sinusoidal signal, the impact signal 1 (amplitude is 0.7, the period is 100, frequency is 20 Hz); impact signal 2 (amplitude is 0.7, the cycle is 33, frequency is 60 Hz); and impact signal 3 (amplitude is 1.0, the cycle is 15.3, frequency is 130 Hz). Our purpose is to extract each fault feature from the simulation signals with multiple impacts. We directly use MOMED for the simulation signal to reduce noise and take different noise-reduction intervals, the result of which is shown in Figure 6, and each time an impact signal is obtained, the effect of noise reduction improves. The final extraction period of different noise-reduction intervals is also different, and the three impact vibrations of the original signal are not extracted. The reason is that the noise-reduction interval of the signal is not set properly. Therefore, the simulation signal needs to be processed by frequency division. The aim is to make sure it has a unique timescale in the IMF.

**Figure 7:**
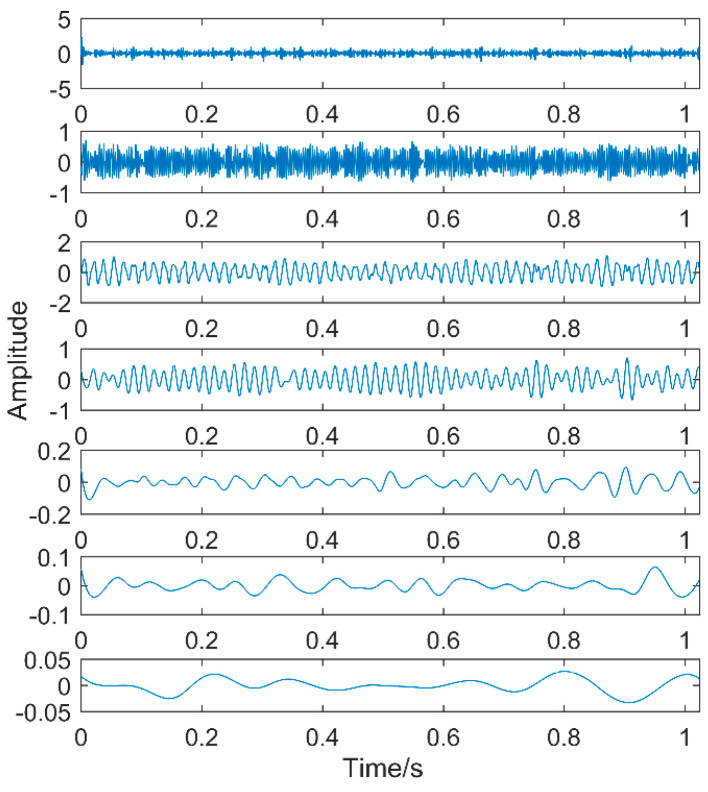
Simulation signal after Ensemble Empirical Mode Decomposition (EEMD) decomposition results.

EEMD decomposition of the simulation signal is shown in Figure 7. 

**Figure 8:**
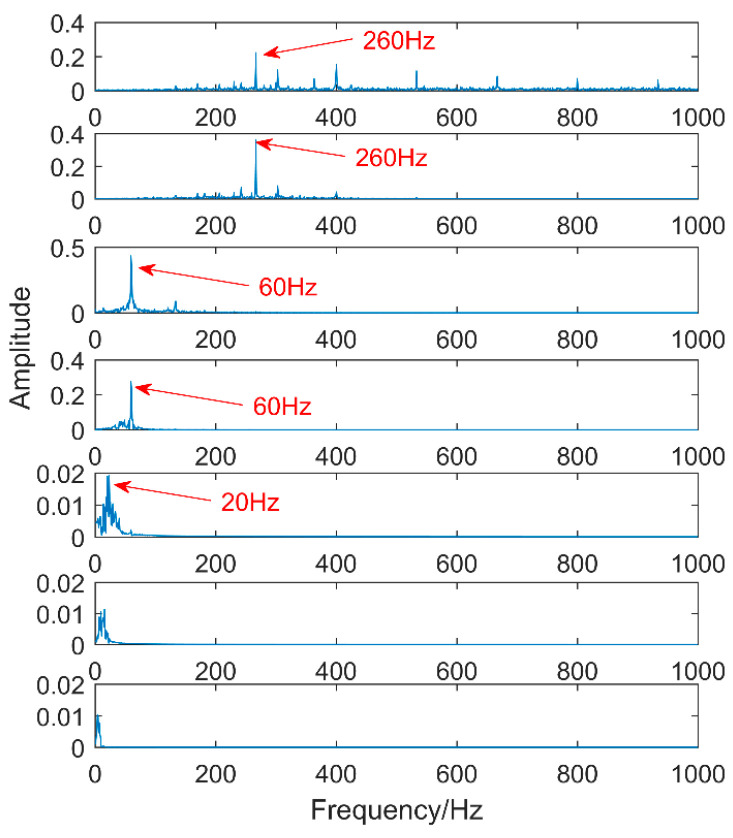
Spectrum of intrinsic mode functions (IMFs) decomposed after EEMD.

The first eight layers of the IMF with strong correlation with the original signal are taken. From the time-domain graph, it can be seen that each layer of the IMF does not contain the three impacts of the original signal. The composition shows that noise still exists in each layer of the IMF. The noise in this area consists of two parts. The first is the noise of the original signal, and the second is that the added white noise is not completely neutralized. Therefore, perform FFT on them separately, as shown in Figure 8.

**Figure 9:**
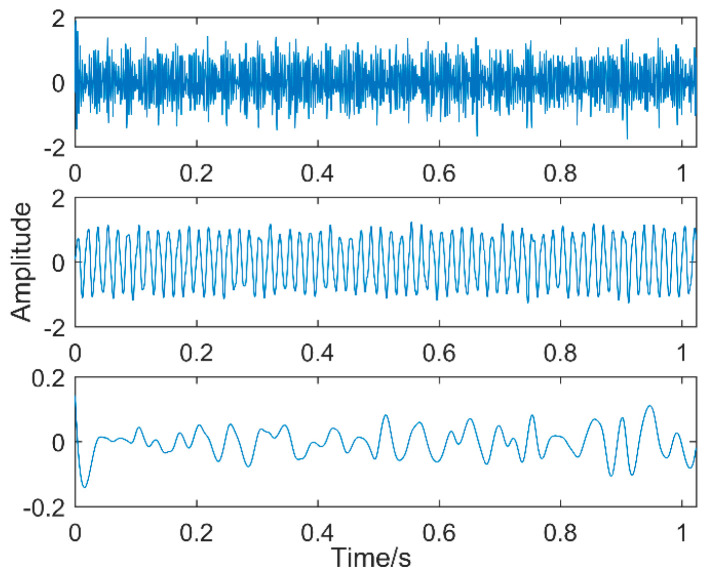
Combined IMF (CIMF) results.

The first two layers contain the same timescale, the third and fourth contain the same scale, and the fifth and sixth layers contain the same scale. Recombine IMFs with the same timescale to obtain CIMF1, CIMF2, CIMF3, as shown in Figure 9.

**Figure 10:**
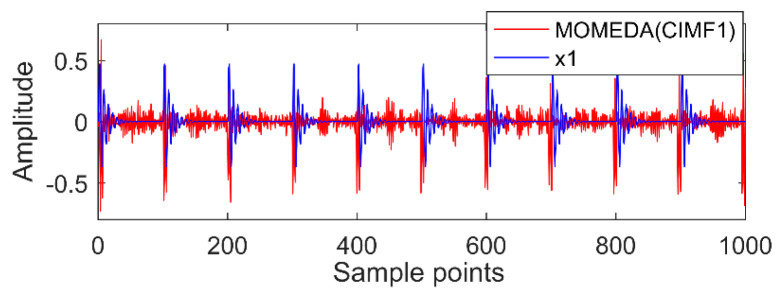
MOMED (CIMF1).

**Figure 11:**
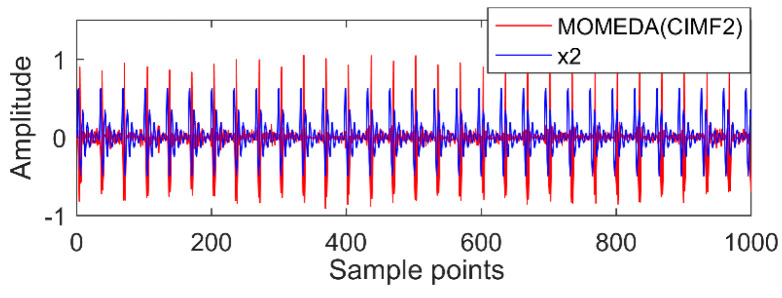
MOMED (CIMF2).

**Figure 12:**
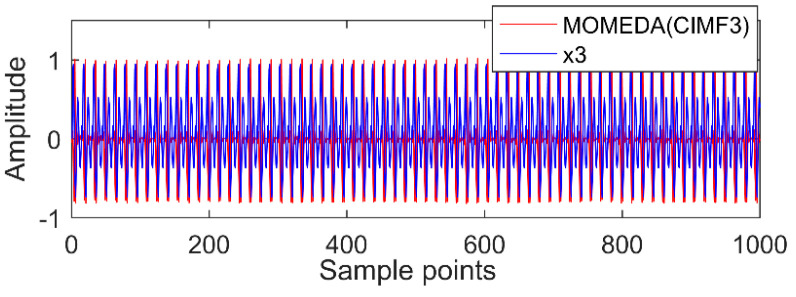
MOMED (CIMF3).

The new IMF contains only a single timescale. It is determined by calculation that their periods are 100, 60, and 15 (sample points), and then the appropriate period intervals are (90, 110), (51, 70), and (11, 19). The results of MOMED noise reduction are shown in Figure 10, Figure 11 and Figure 12. MOMED (CIMF1) indicates that MOMED denoising of CIMF1 and reconstructing X1 of the original signal apparently has extracted the X1 impact component. In addition to strong impact, the noise is still distributed over the entire time domain, but the copy is relatively small without affecting the overall judgment. The noise-reduced signals and original signals of MOMED (CIMF2) and MOMED (CIMF3) are almost completely reconstructed. It is further illustrated that the proposed compound fault feature extraction method has strong engineering application value.

**Figure 13:**
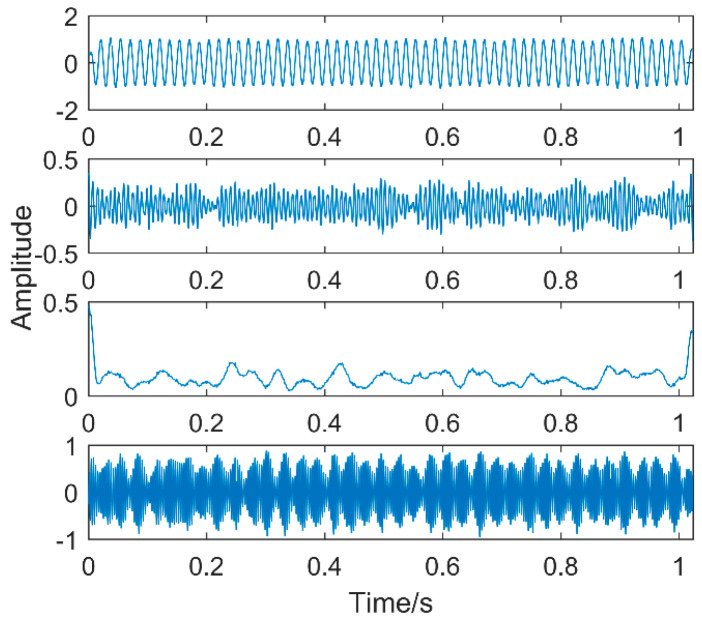
Simulation signal after VMD decomposition results.

**Figure 14:**
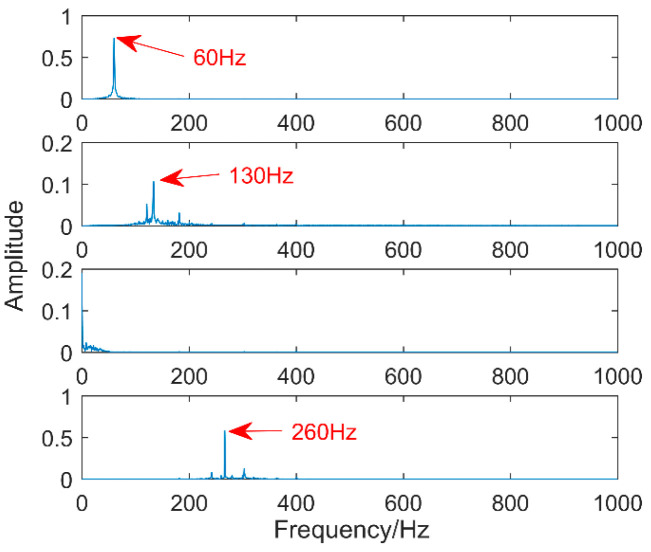
Spectrum of IMFs decomposed after VMD.

In order to compare the effectiveness of the proposed method, the simulation signal is analyzed with VMD. The K = 4 and the penalty factor are 3000. Considering that the penalty factor has little effect on the algorithm, the reason of K = 4 is that the simulation signal contains four frequency components in order to decompose it into four eigenmode functions. The result is shown in Figure 13. The number of decomposed layers is K = 4. The corresponding spectrum is shown in Figure 14, in which the second and fourth layers are shown. Corresponding to the characteristic component 130 Hz, the first layer is 60 Hz, the characteristic component of the third layer can’t determine its composition, and 20 Hz vibration frequency can’t be extracted. Therefore, in a strong-noise environment, VMD extraction fault features are also prone to mode mixing.

## 5. Application Case 

**Figure 15:**
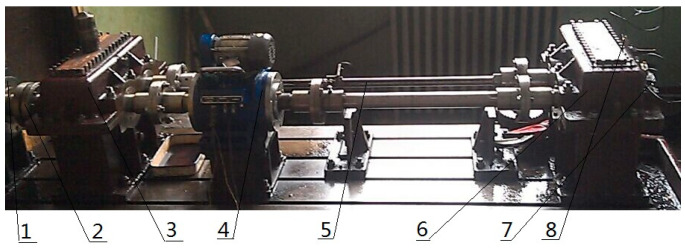
Rig for gear-transmission testing (1: Speed-adjustable motor, 2: coupling, 3: accompanied gearbox, 4: speed-reversing instrument, 5: torsion bar, 6: test gearbox, 7: three-way acceleration sensor 1#, 8: three-way acceleration sensor 2#).

**Figure 16:**
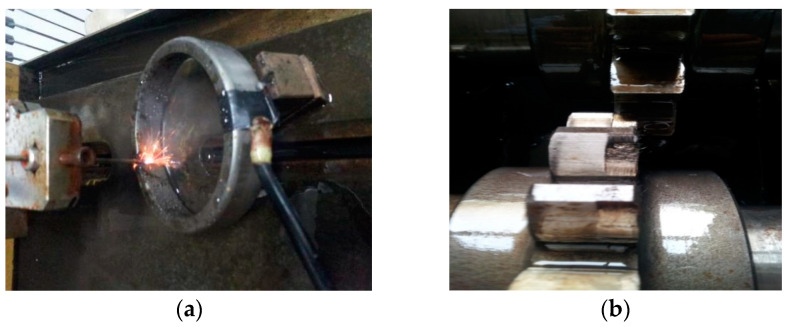
Bearing and gear fault diagram. (**a**) Bearing outer ring defect; (**b**) Gear peeling.

The experimental device mainly includes speed display, three-way acceleration sensors, test gears (18 teeth), test bearings, a motor, shafts, and so on. The type of the test bearing is 32,212, the transmission ratio of the test gear is 1:1, the half-tooth engagement is adopted, the speed is 1200 r/min, and the sampling frequency is 8000 Hz. The test load is carried out step by step, the normal and faulty gears and bearings are loaded to the test load of 1000 N.M, and the type of the three-way acceleration sensor is YD77SA (the sensitivity is 0.01 ${V/\mathrm{ms}}^{2}$). The parameters of the motor are: model 200L-4; voltage 380 V; power 30 KW, constant power frequency conversion is 50–100 Hz; quality is 255 Kg. Fault location including gear peeling, gear outer ring defect (0.2 cm × 0.4 cm). The faulty bearing is located at the three-way acceleration sensor 1#. The fault frequency of the outer ring is 160 Hz, and the number of sampling points is 2048. After a simple calculation, it can be obtained that the fault period of the outer ring is 50, the meshing frequency of the gear is 360 Hz, and the gear meshing cycle is 22. The test rig is shown in Figure 15. The gear fault is pitting, and the outer ring fault is generated by EDM (Electric discharge machining), as shown in Figure 16.

**Figure 17:**
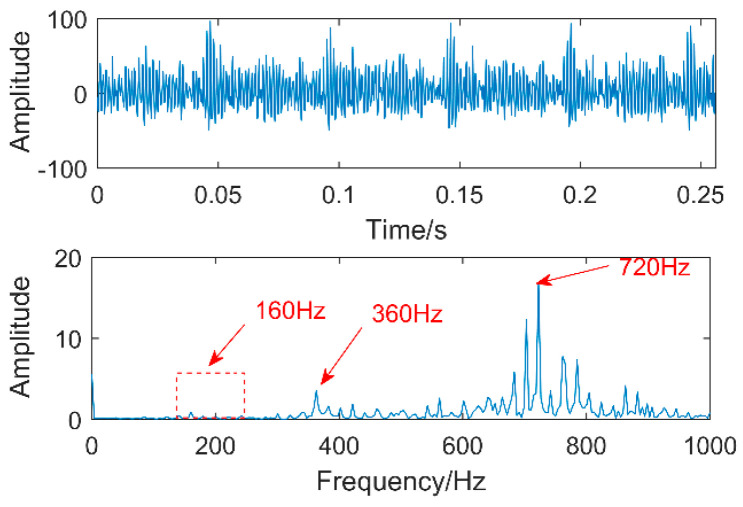
Time domain and spectrum of vibration signals.

**Figure 18:**
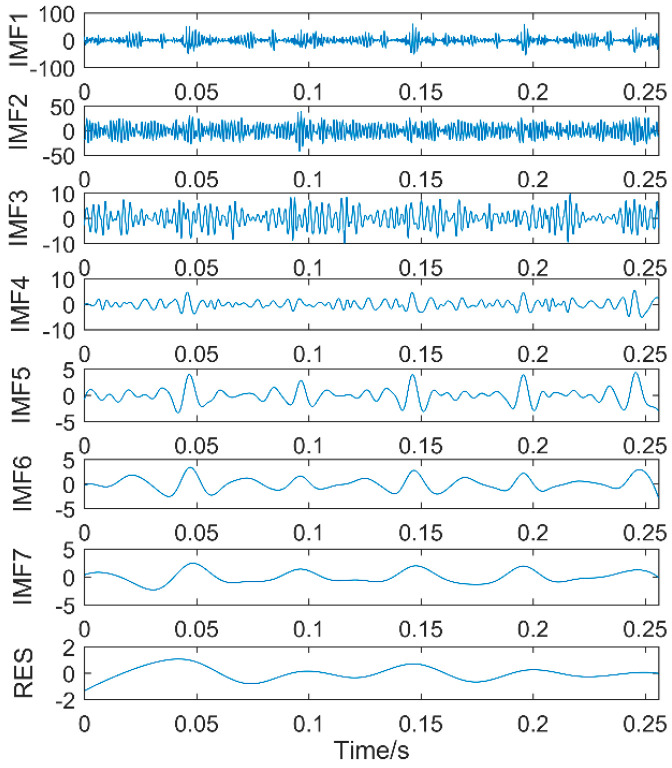
Time domain of vibration signals after EEMD.

**Figure 19:**
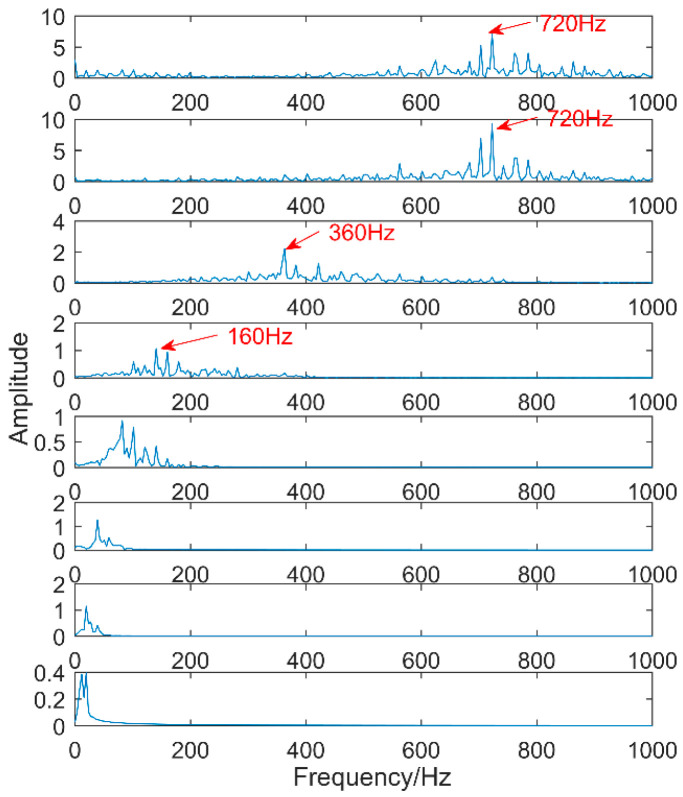
Spectrum of vibration signals after EEMD.

**Figure 20:**
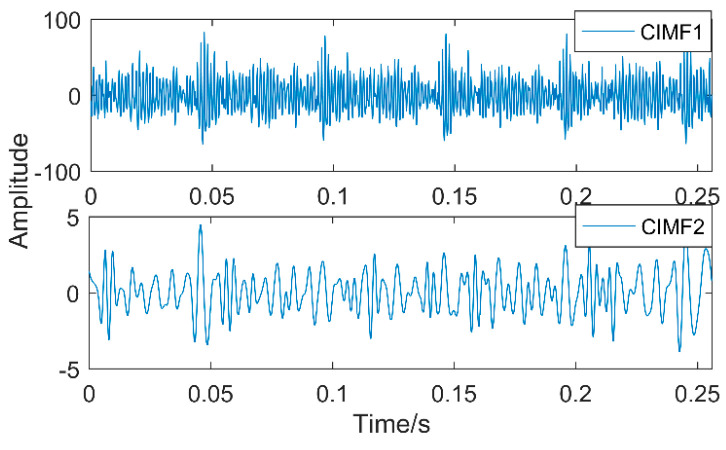
CIMF of vibration signals.

**Figure 21:**
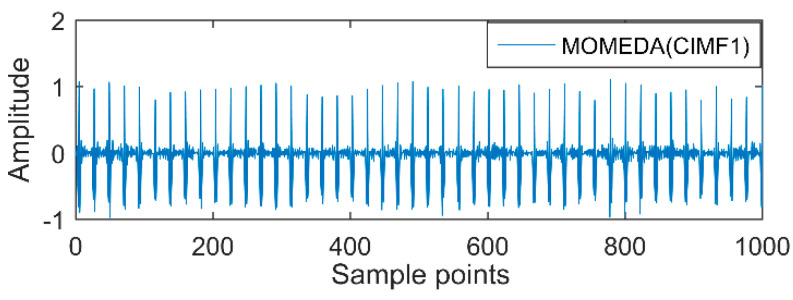
MOMED (CIMF1).

**Figure 22:**
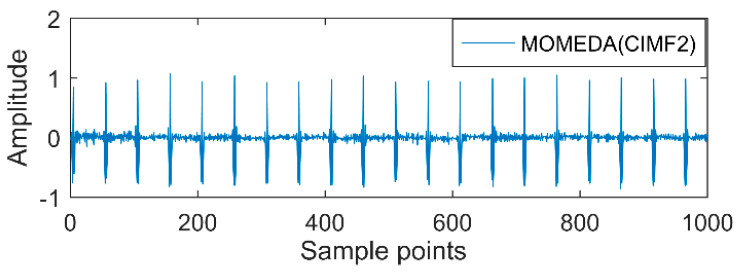
MOMED (CIMF2).

**Figure 23:**
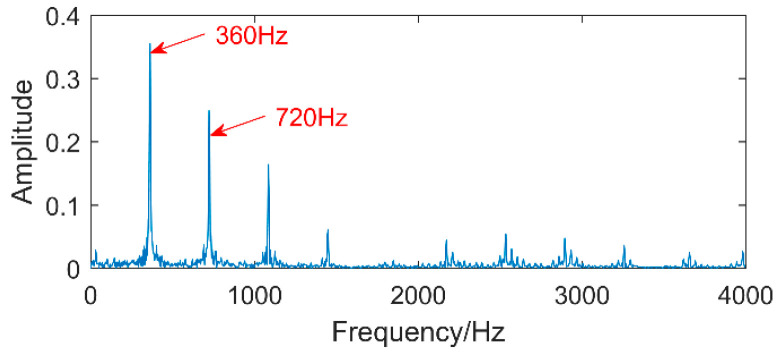
The envelope spectrum of MOMED (CIMF1).

**Figure 24:**
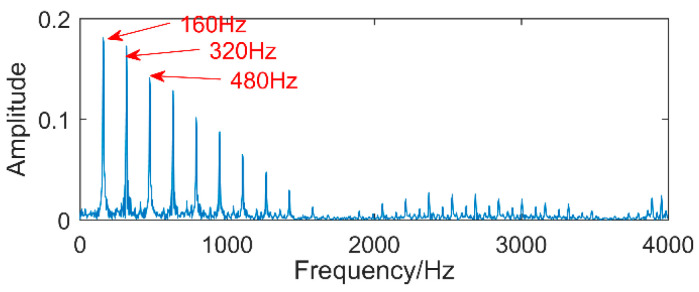
The envelope spectrum of MOMED (CIMF2).

**Figure 25:**
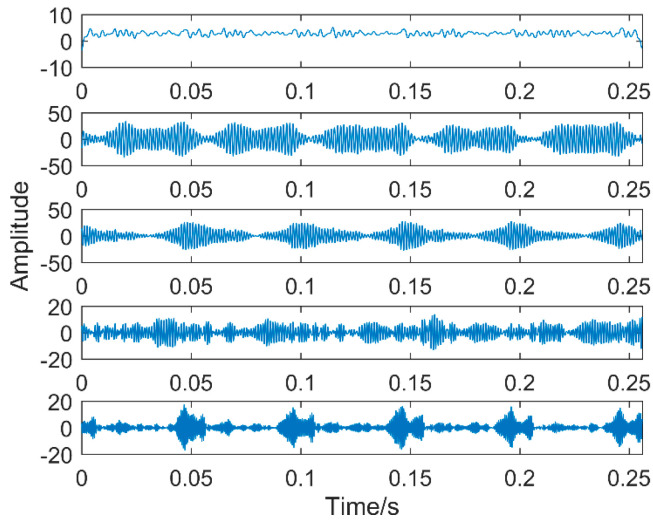
Vibration signals after VMD decomposition results.

**Figure 26:**
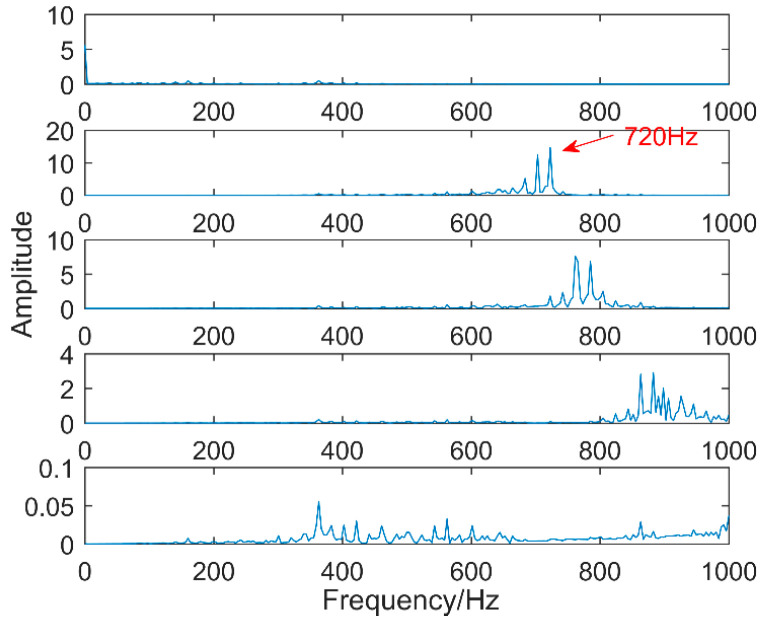
Spectrum of IMFs decomposed after VMD of vibration signal.

The time-domain waveforms of vibration signals of the gearbox measured by the is shown in Figure 17. From the time-domain waveform a significant impact can be seen occurring periodically, corresponding to the gear-meshing frequency; spectral analysis determines this judgment, a small peak at 360 Hz, 720 Hz, and asymmetrical sideband, and there is no peak of the frequency of bearing failure. EEMD is applied to obtain Figure 18, and the corresponding frequency spectrum is shown in Figure 19. The first four layers have the strongest correlation with the original signal. The first three layers have center frequencies of 720 Hz, 720 Hz, and 360 Hz, respectively. These are the same timescale; the center frequency is 160 Hz, and EMMD has obvious mode mixing. Although the fault information of gears and bearings can be determined by the spectrum, mode mixing can easily lead to misdiagnosis or leakage diagnosis, and therefore the energy of the fault information needs to be enhanced. According to the innovation of the article, the first three layers were reorganized to obtain CIMF1, and the fourth layer was CIMF2. The results are shown in Figure 20. The two layers of mode functions contain most of the energy in the original fault signal. Since their periods are 22 and 55, respectively, the noise-reduction interval is set to (15, 24) and (50, 70) in order to extract the periodic information. Respectively obtained MOMED (CIMF1) and MOMED (CIMF2), the results of which are shown in Figure 21 and Figure 22, and continuous periodic impact has been extracted. Each impact entropy is stronger than the EEMD decomposition results. Through envelope analysis, the results are shown in Figure 23 and Figure 24. Their fundamental frequencies are 360 Hz and 160 Hz, respectively, which fully attenuates the noise interference and extracts compound fault features. In order to further compare with the method proposed in the article, VMD is used to analyze the vibration signal. The penalty factor was 2000, and the number of layers for decomposition was 5. The results are shown in Figure 25. The corresponding frequency spectrum is shown in Figure 26. Only 720 Hz is the corresponding fault information. Since each layer ratio must have a center frequency when the VMD is decomposed, mixing occurs in other layers. In addition, the number of decomposition levels of the VMD needs to be determined artificially. Therefore, the results of the decomposition are indefinite and they are prone to misdiagnosis or missed diagnosis.

## 6. Conclusions

(1) The decomposition results by the EEMD method are related to the added white noise with a noise level, which results in the degradation of decomposition accuracy. Under a strong-noise environment, the artificially determined white noise cannot decompose fault signals with different features into different IMFs. On the contrary, the same fault feature may be decomposed into several layers of IMFs, resulting in entropy leakage.

(2) The accuracy of MOMED noise reduction is determined by the noise-reduction interval. If you choose improperly, you will miss the diagnosis. MOMED can only extract a single fault message at a time. EEMD not only adaptively separates the original signal in order of high and low frequencies, but also is an adaptive filter. This article can improve the entropy of the same fault feature by combining the IMFs of the same timescale. In different timescales, setting different noise-reduction intervals can extract complex fault features.

(3) The validity of the IMOMED method is proved by the simulation signal and the measured signal, and the compound fault features can be extracted successfully by the proposed method, which has immunity even under strong background noise.

(4) The advantage of this article is to improve the SNR by EEMD, overcome the modal aliasing phenomenon by combining the modal functions, and then extract the impact components with MOMED. The disadvantage is that the adaptation of the method proposed in the paper needs further analysis and discussion.
